# The role of oral and pharyngeal motor exercises in post-stroke recovery: A scoping review

**DOI:** 10.1177/02692155221141395

**Published:** 2022-11-25

**Authors:** Reeman Marzouqah, Anna Huynh, Joyce L Chen, Mark I Boulos, Yana Yunusova

**Affiliations:** 1 Rehabilitation Sciences Institute, 7938 University of Toronto, Toronto, ON, Canada; 2 Hurvitz Brain Sciences Research Program, Sunnybrook Research Institute and Sunnybrook Health Sciences Centre, Toronto, ON, Canada; 3 KITE, University Health Network, Toronto, ON, Canada; 4 Faculty of Kinesiology and Physical Education, 7938 University of Toronto, Toronto, ON, Canada; 5 Department of Medicine, Division of Neurology, 7938 University of Toronto, Toronto, ON, Canada

**Keywords:** stroke, exercise, muscles, oral cavity, pharynx

## Abstract

**Objective:**

To analyze intervention goals, protocols, and outcome measures used for oral and pharyngeal motor exercises in post-stroke recovery.

**Data sources:**

MEDLINE, EMBASE, CINAHL, PsychINFO, and Cochrane databases were searched in September 2022.

**Methods:**

Studies were included if they (1) recruited post-stroke adult patients, (2) administered exercises for the oral and/ or pharyngeal muscles, and (3) reported results at baseline and post-exercise. The extracted data included intervention goals, protocols, and outcomes. All outcomes were classified according to the International Classification of Functioning, Disability and Health (ICF).

**Results:**

A total of 26 studies were identified. Their intervention goals aimed to rehabilitate a broad spectrum of muscle groups within the oral cavity and pharynx and to improve the functions of swallowing, speech, facial expressions, or sleep breathing. Protocol duration ranged from 1 to 13 weeks, with various exercise repetitions (times per day) and frequency (days per week). Half of the studies reported using feedback to support the training, and these studies varied in the feedback strategy and technology tool. A total of 37 unique outcome measures were identified. Most measures represented the body functions and body structure component of the ICF, and several of these measures showed large treatment effects.

**Conclusions:**

This review demonstrated inconsistency across published studies in intervention goals and exercise protocols. It has also identified current limitations and provided recommendations for the selection of outcome measures while advancing a multidisciplinary view of oral and pharyngeal exercises in post-stroke recovery across relevant functions.

## Introduction

Motor impairments in the oral cavity and pharyngeal musculature can cause dysphagia, a swallowing disorder,^1^ or dysarthria, a motor speech disorder^2^ in up to 40% of individuals who experienced a stroke.^3^ Likewise, central facial palsy is common post-stroke, affecting individuals’ ability to communicate and express themselves emotionally.^4^ Further, weakness and hypo-activation of the oropharyngeal muscles are linked to highly prevalent post-stroke obstructive sleep apnea.^5^ Although there is strong evidence that exercise interventions after stroke restore strength, accuracy, range, and/or speed of motion of the impaired musculature^6^ and have a beneficial role in improving cardiovascular fitness,^7^ gait,^8^ and upper limb function,^9^ the evidence is still emerging regarding the use of oral and pharyngeal motor exercises targeting relevant muscles to improve swallowing, speech, facial and sleep-breathing functions.^10,11^

Several previous reviews summarized the evidence for the use of oral and pharyngeal motor exercises for participants without stroke or mixed clinical populations.^12–17^ These reviews were further limited to a single function, for example, swallowing,^14,16,17^ speech,^15^ sleep-related breathing,^12^ or facial expressions.^13^ However, patients typically do not present in rehabilitation clinics with an isolated dysfunction. Twenty-eight percentage of patients with dysarthria demonstrate dysphagia,^3^ and between 16% and 78% of those with obstructive sleep apnea, depending on a study, will have dysphagia.^18^ Despite this, the clinical practice remains siloed, and a multidisciplinary lens in research is not commonplace.^19^ Here, we aimed to review the literature across all related functions, to explore the relationship between exercise intervention effects and each function.

Despite the wide use of oral and pharyngeal motor exercises in post-stroke recovery,^20^ there is no clear understanding regarding which exercises, when and to what extent are beneficial, and there is no clarity on exercise frequency, repetition, and protocol duration.^11,20^ Furthermore, the selection of outcome measures in the context of these exercises remains poorly understood. Yet, the results generated from intervention studies can only be meaningful if the outcomes are appropriately chosen.^21^ The International Classification of Functioning, Disability and Health^22^ has been used to select the most appropriate measures for a research or clinical context.^23,24^ This current review took a systematic approach by classifying outcomes according to the framework and exploring the relationship between measure types and intervention results.

The following questions were asked: (1) What intervention goals have been addressed using oral and pharyngeal motor exercises in post-stroke recovery? (2) What kind of exercise protocols have been implemented? (3) What outcomes have been used? and (4) Are there differences in the results reported by types of outcomes and by the targeted function?

## Methods

A scoping review was conducted following the Arksey and O’Malley (2005)^25^ methodological framework, that was advanced by Levac (2010).^26^ The framework included five major stages: (1) Articulating the research question, (2) Identifying the relevant studies, (3) Selecting studies, (4) Charting the data, and (5) Collating, summarizing, and reporting the results. A scoping review methodology was chosen because the objective was broad and descriptive in nature, that is, to summarize the range of evidence available, identify gaps and challenges, and propose possible solutions.

A search strategies map and detailed search queries were created for five electronic databases: MEDLINE, Embase, CINAHL, PsychINFO, and Cochrane. Searches were conducted in each database from 1980 until September 2022. Search included the use of subject headings (e.g. Medical Subject Headings (MeSH), Entree) and text words including (1) Stroke (e.g. stroke/cerebrovascular accident) (2) Exercise training (e.g. exercise therapy/myofunctional therapy/oro* ADJ4 facial) and (3) Oral/ pharyngeal motor function (e.g. facial droop/tongue/lip*, pharynx*). Searches were limited to peer-reviewed studies published on human adults in the English language. Additional sources included hand searches of references and forward citation tracking of all relevant articles.

The selection of studies for inclusion in the review was conducted in two steps: title and abstract screening, followed by a full-text review of those articles included after the initial screening. Studies were included if they met the following criteria: (1) population of post-stroke individuals over the age of 18 years, (2) administered exercises for the oral and/ or pharyngeal muscles, (3) reported results of at least baseline and post-exercise measures, and (4) had a peer-reviewed full-text in the English language. To synthesize the highest possible quality of evidence, only randomized controlled trials were included in this review.

**Figure 1.**
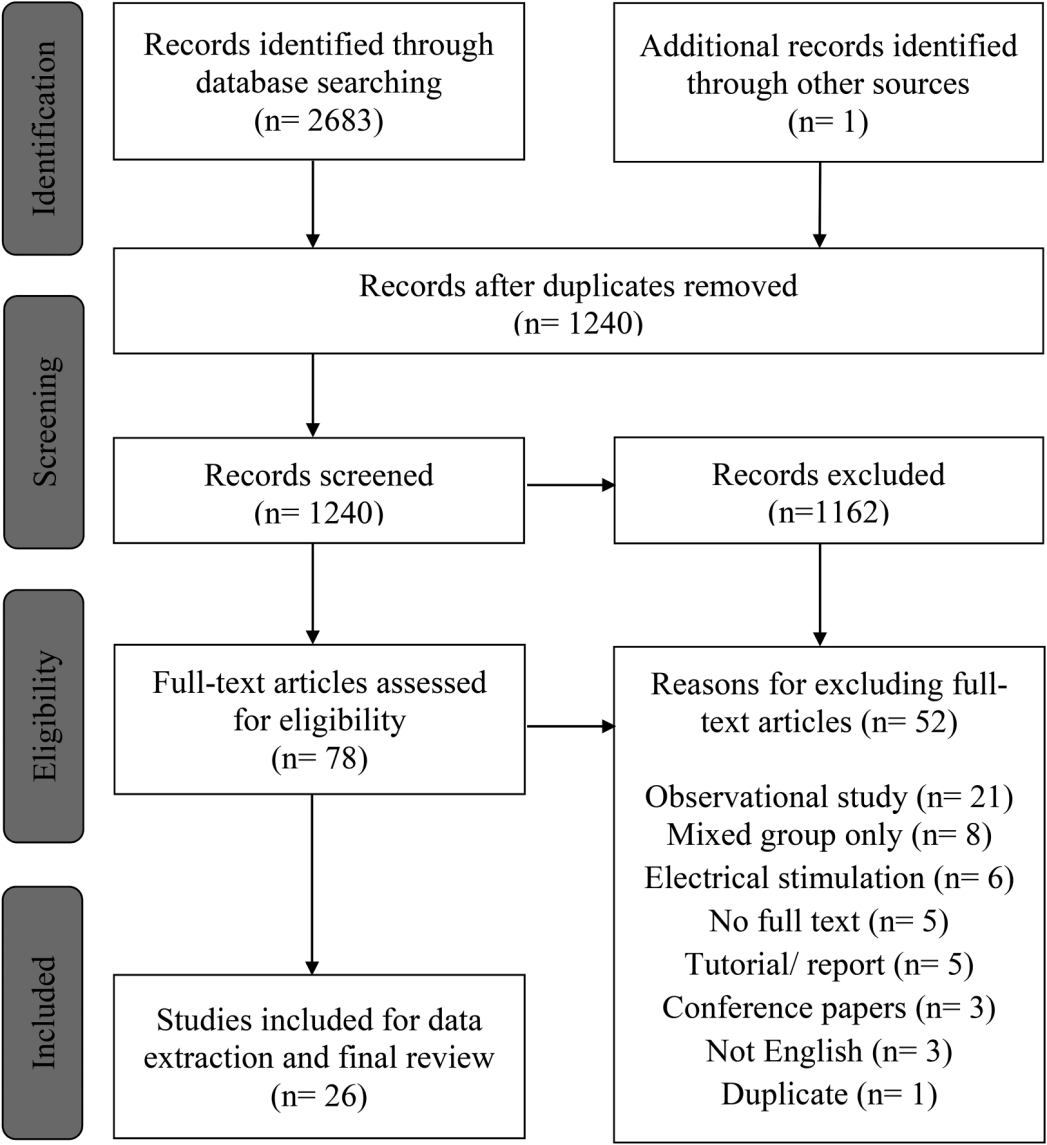
A preferred reporting item for systematic reviews and meta-analyses extension for scoping reviews (PRSIMA-ScR) flow diagram showing the phases of the selection process and the number of records identified, included, and excluded.

The initial search yielded a total of 2683 records, of which 1443 were duplicates (see Figure 1). After removing the duplicates, a single reviewer (RM) assessed all retrieved records’ titles and abstracts and determined their eligibility for potential inclusion. Seventy-eight records were imported into Covidence systematic review software^27^ for full-text review. Two reviewers (RM and AH) screened full-text citations against the eligibility criteria. All articles deemed eligible by any of the reviewers were independently assessed for inclusion by the other reviewer. Fifty-two articles were excluded for various reasons listed in Figure 1.

Two authors (RM and AH) developed a data charting form and extracted data from the accepted full-text articles. At the onset of data charting, a pilot was conducted on three full-text articles, and reviewers met to discuss responses to establish consistency. The form included data on study characteristics, such as first author, year, sample size and subjects’ demographics. To address our research questions, we also included the following data: (1) intervention goals, which contain the global function (e.g. swallowing), muscle groups (e.g. tongue muscles) and physiological goal/s (e.g. strength), (2) elements of protocol design (e.g. use of technology tools, feedback, schedule, setting), (3) all outcome measures used (i.e., primary, and secondary, motor and non-motor), and (3) results that included statistical analyses of outcome measures.

Two authors (RM and AH) classified the type of measures using definitions of the International Classification of Functioning, Disability and Health components—(1) body functions and body structure, (2) activities and participation, and (3) contextual (environmental) factors. If discrepancies were noted in the linking process, discussion and consensus decision were reached. The International Classification of Functioning, Disability and Health definitions related to oral and pharyngeal motor exercises are presented in Table 1.^22^

**Table 1.** Definitions of the international classification of functioning, disability, and health.^22^

Components of the international classification of functioning, disability, and health	Relevant chapters in the international classification of functioning, disability, and health
**Body functions and body structure:** Physiological processes and anatomical parts of the body	**B1 Mental function:** Global and specific functions of the brain, including sleep **B3 Voice and speech functions:** Functions of producing sounds and speech **B4 Functions of the cardiovascular, hematological, immunological, and respiratory systems.** Functions of the heart, blood, immunity, and respiration **B5 Functions of the digestive, metabolic, and endocrine systems:** Functions of swallowing, digestion, and endocrine glands **B7 Neuromusculoskeletal and movement-related functions:** Functions of muscles, joints, bones, and reflexes **B730-749 Muscle functions:** Functions related to muscle force, tone, and endurance **B760 Voluntary movement functions:** Functions associated with control and coordination of voluntary movements **S3 Structures involved in voice and speech:** Structures of the mouth, pharynx, and larynx
**Activities and participation:** Execution of tasks that interplay multiple functions and structures	**D3 Communication:** Producing and receiving language **D4 Mobility:** Transferring from one place to another **D5 Self-care:** Caring for oneself **D7 Interpersonal interaction and relationships:** Actions and tasks required for basic and complex interactions **D8 Major life areas:** Actions and tasks required to engage in education and work **D9 Community, social and civic life:** Actions and tasks required to engage in social and civic areas of life
**Environmental Factors:** Physical, social and attitudinal context in individual's environment	**E1 Products and technology:** Products or technology adapted for improving the functioning of a person

To explore the association between outcome measure types and results, the effect sizes (Cohen's *d*)^28^ between the two study groups (i.e. experimental and control) were extracted. Studies in which the control group did not undergo standard care or sham therapy were excluded from this analysis. We operationalized a small effect as *d* ≥ 0.2, a medium effect as *d* ≥ 0.5, and a large effect as *d* ≥ 0.8.^28^ Computations and plots were conducted using the “*metafor”* package^29^ in R, version 4.0.2.^30^ The effect sizes were not combined for meta-analysis, as the purpose of this scoping review was to explore the descriptive differences in the results by the outcome type and by function, instead of focusing on the efficacy of interventions.

## Results

A total of twenty-six^31–57^ randomized controlled trials reporting oral and pharyngeal motor exercise interventions during post-stroke recovery were identified (see Table 2). Two articles which analyzed data from the same sample (McCullough et al., 2012, 2013)^54,55^ were considered as one study in this review.

**Table 2.** Characteristics of studies and participants in the review.

Study	Study design	Experimental group	Control group	Sex M/F	Age years	Time since stroke	Stroke type	Stroke lesion
**2022 Nordio^57^**	Pilot randomized controlled trial	Swallowing exercises with surface electromyography, *n*=9	Swallowing exercises without surface electromyography, *n*=7	12/4	71 ± 9.7 69 ± 11.7	Subacute	Ischemic, hemorrhagic	Supratentorial, infratentorial, both
**2020 Carnaby^33^**	Randomized controlled trial	Swallowing exercises (“McNeil program”), *n* = 18	Usual care, *n* = 17	15/20	70.6 ± 11.8 64.3 ± 14.7	Acute, subacute	Ischemic, hemorrhagic	Supratentorial, infratentorial
**2020 Choi^32^**	Pilot randomized controlled trial	Jaw opening exercise, *n* = 11	Head-lift exercise, *n* = 10	9/12	63.5 ± 7.7 61.2 ± 9.7	Subacute	Ischemic, hemorrhagic	Right, left
**2020** **Park^31^**	Randomized controlled trial	Jaw opening exercise, *n* = 15	Sham exercises, *n* = 14	17/12	62.1 ± 10.1 61.8 ± 12.1	Subacute	Ischemic, hemorrhagic	Right, left
**2019 Hwang^35^**	Randomized controlled trial	Tongue stretching exercises, n = 11	Thermal-tactile stimulation techniques and swallowing maneuvers, *n* = 10	11/10	60.5 ± 12.5 62.2 ± 10.3	Subacute	Ischemic, hemorrhagic	Right, left
**2019** **Kim^36^**	Randomized controlled trial	Modified chin-tuck against resistance exercise, *n* = 12	Thermal-tactile stimulation techniques, *n* = 13	12/13	63.5 ± 5.5 65.2 ± 6.2	Chronic	Ischemic, hemorrhagic	Right, left
**2019 Moon^34^**	Randomized controlled trial	Orofacial muscle exercises on smartphone, *n* = 8	Orofacial muscle exercises on paper, *n* = 8	9/7	54.1 ± 5.4 55.3 ± 14.8	Subacute	Ischemic, hemorrhagic	Supratentorial, infratentorial
**2019a Park^37^**	Randomized controlled trial	Effortful swallowing exercise, *n* = 12	Placebo therapy of saliva swallow, *n* = 12	11/13	66.5 ± 9.5 64.8 ± 11.2	Chronic	Not reported	Supratentorial, infratentorial
**2019b Park^38^**	Randomized controlled trial	Chin-tuck against resistance, *n* = 20	Head-lift exercise, *n* = 20	23/17	60.9 ± 11.1 59.4 ± 9.3	Chronic	Ischemic, hemorrhagic	Right, left
**2018** **De Fraga^39^**	Pilot randomized controlled trial	Myofunctional exercises without vocal exercises, *n* = 5	Myofunctional exercises with vocal exercises, *n* = 5	Not reported	73.2 ± 7.6 63.8 ± 12.9	Not reported	Ischemic	Not reported
**2018 Moon^40^**	Pilot randomized controlled trial	Tongue strengthening exercise with accuracy training, *n* = 8	Thermal-tactile stimulation techniques, swallowing maneuvers and diet modification, *n* = 8	7/9	62 ± 4.1 63.5 ± 6.5	Subacute	Ischemic, hemorrhage	Supratentorial, infratentorial
**2018** **Ye^56^**	Randomized controlled trial	Oropharyngeal muscle exercises, *n* = 25	Placebo therapy of deep breathing exercise, *n* = 24	36/13	63.4 ± 9.9 65.5 ± 10.4	Subacute	Ischemic, hemorrhagic	Not reported
**2018 Park^41^**	Randomized controlled trial	Chin-tuck against resistance exercise, *n* = 11	Thermal-tactile stimulation techniques and swallowing maneuvers, *n* = 11	10/12	62.1 ± 17.2 58.4 ± 12.5	Chronic	Ischemic, hemorrhage	Supratentorial
**2017 Kang^42^**	Randomized controlled trial	Orofacial muscle exercises, *n* = 10	Orofacial exercises with mirror effect, *n* = 11	13/8	63.1 ± 10.3 55.6 ± 16	Subacute	Ischemic, hemorrhagic	Not reported
**2017 Choi^43^**	Randomized controlled trial	Shaker exercise, *n* = 16	Thermal-tactile stimulation techniques and swallowing maneuvers, *n* = 15	19/12	60.8 ± 10.8 60.4 ± 10.5	Chronic	Ischemic, hemorrhagic	Right, left
**2017 Koyama^53^**	Pilot randomized controlled trial	Modified jaw opening exercise, *n* = 10	Sham therapy of jaw closing exercise, *n* = 2	10/2	66.0 ± 9.3 71.8 ± 7.6	Subacute	Not reported	Supratentorial, infratentorial
**2017 Moon^45^**	Randomized controlled trial	Tongue strengthening exercise, *n* = 8	Physical and occupational therapy, *n* = 8	11/5	64.7 ± 5.7 62.5 ± 5.9	Chronic	Ischemic, hemorrhagic	Right, left
**2017 Park^46^**	Randomized controlled trial	Head-lift exercise, *n* = 13	Thermal-tactile stimulation techniques and swallowing maneuvers, *n* = 14	17/10	59.2 ± 11.9 61.5 ± 13.6	Chronic	Ischemic, hemorrhagic	Supratentorial, infratentorial
**2017** **Gao^47^**	Randomized controlled trial	Chin-tuck against resistance, *n* = 30	Swallowing therapy (not specified), *n* = 30, Shaker exercises, *n* = 30	42/48	70.8 ± 6.6 71.1 ± 7.0 71.1 ± 6.4	Acute, subacute	Ischemic	Not reported
**2017** **Kim^44^**	Randomized controlled trial	Tongue strengthening exercise, *n* = 18	Thermal-tactile stimulation techniques and swallowing maneuvers, *n* = 17	19/16	62.1 ± 11.0 59.2 ± 10.1	Chronic	Ischemic, hemorrhagic	Right, left
**2016 Steele^48^**	Randomized controlled trial	Tongue strengthening protocol with saliva swallow, *n* = 6	Tongue strengthening protocol without saliva swallow, *n* = 5	7/4	67 ± 16 74 ± 11	Subacute, chronic	Ischemic, not known	Right, left
**2016 Byeon^49^**	Randomized controlled trial	Orofacial myofunctional exercises, *n* = 23	Thermal-tactile stimulation techniques, *n* = 25	14/34	62.5 ± 6.5 64 ± 7.1	Chronic	Not reported	Not reported
**2015 Park^50^**	Randomized controlled trial	Tongue strengthening exercise, *n* = 15	Swallowing therapy (not specified), *n* = 14	13/16	67.3 ± 10.6 65.8 ± 11.5	Chronic	Ischemic, hemorrhagic	Not reported
**2014 Konecny ^51^**	Randomized controlled trial	Orofacial therapy, *n* = 50	Communication therapy, *n* = 49	53/46	53 ± Not reported /60 ± Not reported	Subacute	Not reported	Not reported
**2014 Mackenzie^52^**	Pilot randomized controlled trial	Non-speech oro-motor exercise, *n* = 20	Articulation and prosody exercises, *n* = 19	16/13	Not reported	Chronic	Ischemic, hemorrhagic, not known	Supratentorial, infratentorial, mixed, not known
**2012 and** **2013 McCullough^54,55^**	Pilot crossover randomized controlled trial	Mendelsohn maneuver exercise, *n* = 9	No intervention, *n* = 9	11/7	Not reported	Chronic	Ischemic, hemorrhagic,	Right, left Bilateral, not known

In all studies, the experimental group engaged in an exercise program that targeted at least one muscle in the oral cavity and/or pharynx. The control intervention typically consisted of a standard of care (e.g. swallowing therapy, communication therapy) or sham therapy. Seven studies provided an alternative exercise protocol (e.g. Head-lift exercise).^32,34,38,39,47,48,57^ Sample sizes ranged from 10 to 99 patients and included both sexes. Across studies, the average age of stroke survivors ranged from 53 to 72.5 years. Most studies included people in the subacute (seven days to six months^58^) and chronic (beyond six months^58^) stages post-stroke. Participants were of mixed stroke types (ischemic or hemorrhagic) with lesions in various regions.

Table 3 summarizes the intervention goals in the context of functions, muscle groups, and physiological goals. All studies focused on strengthening at least one oral or pharyngeal muscle. Other physiological goals, that is speed, range of motion, or coordination, were less frequently addressed. Regardless of the physiological goal or muscle group, studies aimed to improve the function of swallowing, speech, facial expressions, and sleep breathing, but the great majority of studies were focused on improving swallowing function (*n* = 21).

**Table 3.** Intervention goals.

Function	Muscle group	Physiological goal
Swallowing ***n* = 21**	Pharyngeal/ laryngeal ***n* *=* 14**	Strength^31–34,36,38,39,41,43,46,47,53,55,57^
	Tongue ***n* *=* 10**	Strength^34,37,39,40,44,48,49,57^, range of motion^35,39^, speed^49^**,** coordination^49,57^
	Lip ***n* *=* 3**	Strength^34,39,49^**,** range of motion^39^, speed^49^**,** coordination^49^
	Cheek ***n* *=* 3**	Strength^34,39,49^
	Jaw ***n* *=* 1**	Strength^49^**,** speed^49^**,** coordination^49^
	Palate ***n* *=* 1**	Strength^39^
Speech ***n* = 2**	Tongue ***n* *=* 2**	Strength^45^, range of motion^52^**,** speed^52^
	Lips ***n* *=* 1**	Strength^52^, range of motion^52^^,^ speed^52^
Facial expressions ***n* *=* *2***	Lips ***n* *=* 2**	Strength^42,51^^,^ range of motion^42,51^
Sleep breathing ***n* *=* 1**	Pharyngeal ***n* *=* 1**	Strength^56^

Pharyngeal/laryngeal muscles were the most frequently targeted overall (*n* = 14). The tongue was the next most targeted muscle group (*n* = 10). Lip and cheek muscles were targeted in six studies. Studies rarely targeted the lower jaw and palatal muscles.

**Figure 2.**
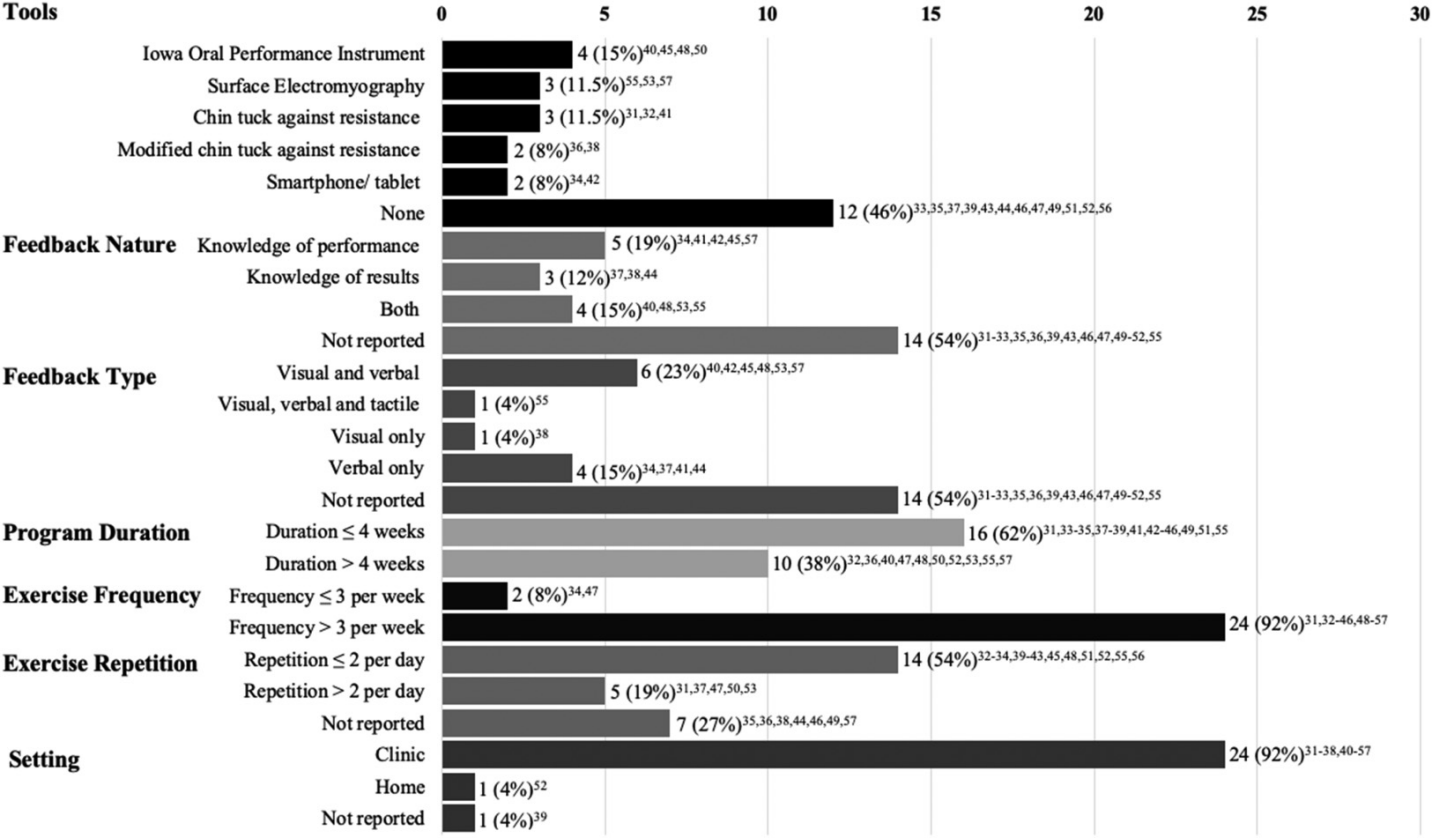
Count of studies by protocol elements.

There was a large variation across protocol designs (see Figure 2). Fourteen studies reported using technology tools to support the training and/or provide feedback. The rest of the studies did not use tools and instead provided exercise instructions verbally or via paper.

The use of feedback was not explicitly described in over half of all studies. Studies that reported using feedback, employed it in the form of knowledge of performance, knowledge of results, or combined. Feedback was often provided visually by displaying information on a device/ tool about the movement pattern or pressure generated during resistance training. In most studies, visual feedback was supported by verbal feedback from the instructor. In addition to more typical visual and verbal feedback, one study employed internal tactile feedback (i.e. patients palpated their skin to feel the movement on their neck).

The program duration ranged from 1 to 13 weeks, with many studies administering exercises twice a day for at least 3 weeks. Exercises were typically completed in the clinic. Only one study employed self-directed home training with some form of guidance at the beginning of the program.

**Figure 3.**
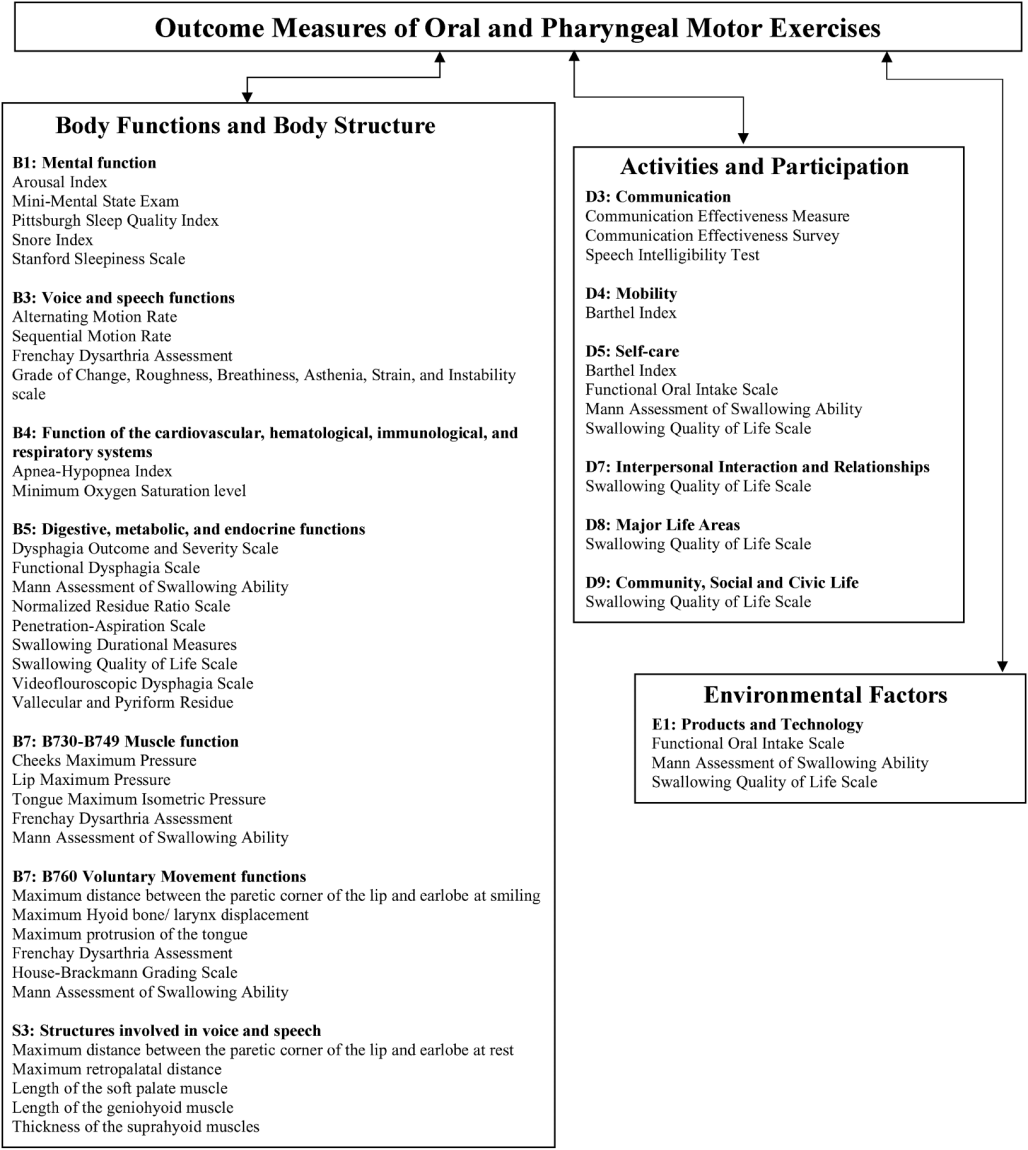
The classification of outcome measures according to the international classification of functioning, disability, and health.

A total of 72 outcome measures have been used across studies. Of these, 37 unique outcome measures were identified and categorized by the International Classification of Functioning, Disability and Health (see Figure 3). Out of the 37 measures, 30 represented the body functions and body structure component, seven—the activities and participation, and three—the environmental factors component. Measures such as, the Mann Assessment of Swallowing Ability and Functional Oral Intake Scale had items related to “Food” and “assistive product and technology for personal use in daily living” and as such were classified under “Environmental factors.”

Within the body functions and body structure categories, B1 to B5 chapters represented “global” functions of thinking, speaking, swallowing, and breathing, B7 represented muscle/movement function, and S3 represented structures in the oral cavity and pharynx. Twenty measures revealed the global functions, whereas only nine were linked to the specific muscle/movement functions and five to muscle structure. In the activities and participation component, the most prevalent chapters were D3—communication (*n* = 3) and D5—self-care (*n* = 4). All environmental factors measures were represented by E1-products and technology.

**Figure 4.**
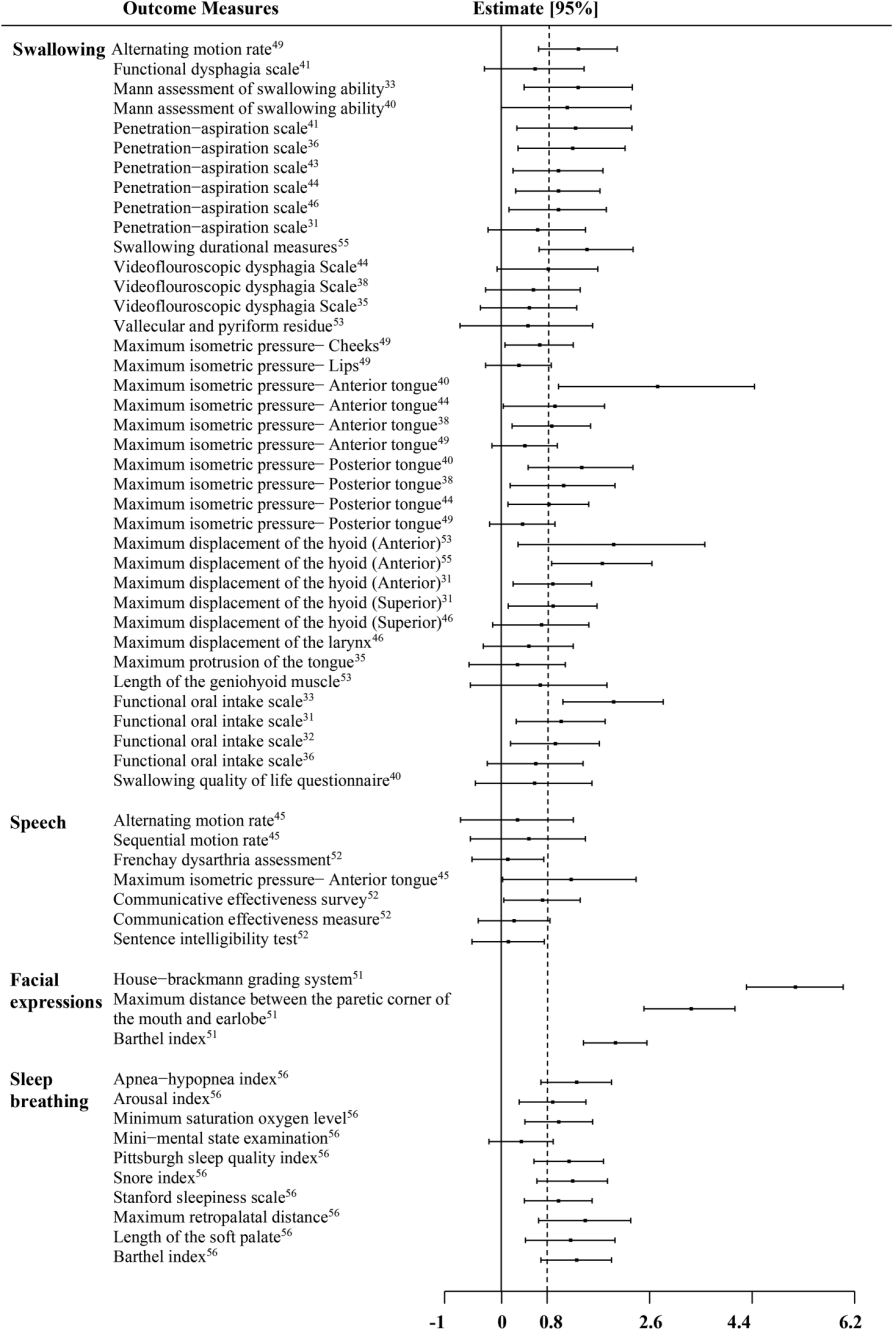
Effect sizes of outcome measures across all studies.

Effect sizes between intervention groups and/or summary data from which the Cohen's *d* and 95% confidence intervals could be extracted were available for a total of 58 outcomes. Figure 4 shows a forest plot, where each bar represented one of these measures. They are ordered by function and sorted by the International Classification of Functioning, Disability and Health components (i.e. starting with body functions and body structure, then activities and participation, and the environmental factors). The review of the outcomes by function revealed 59.5% of measures with large effect sizes in the swallowing category; 14.3% in speech, 100% in facial expression, and 90% in sleep breathing. Among swallowing outcomes, a large treatment effect was noted in 62.5% (10/16) of “global” function measures (e.g. penetration aspiration scale), 66.6% (8/12) of muscle function measures (e.g. maximum isometric pressure), and 66.6% (6/9) of movement function measures (e.g. maximum displacement). Among speech outcomes, the only measure with a large treatment effect—the maximum isometric pressure—anterior tongue—was in B7 (muscle function) category. Facial expressions and sleep breathing studies revealed potentially much stronger results; all three measures used in the facial expressions studies had large effect sizes. All outcomes in sleep breathing studies—except the Mini-Mental State Examination, a test for cognitive impairment—also showed large treatment effects.

## Discussion

This review sought to evaluate the intervention goals, protocols, and outcomes of oral and pharyngeal exercise interventions in post-stroke rehabilitation, in order to identify gaps in the literature and present considerations for the appropriate use of outcomes and advocate for the multidisciplinary approach to post-stroke care.

The findings of this review showed that exercises were used mostly to improve swallowing function, targeting a broad spectrum of relevant muscle groups and physiological goals. All studies in this review prescribed strengthening exercises, performed with or without specialized tools. Exercises not employing tools or targeting physiological goals other than strength (i.e., range of motion and coordination) were typically not well-defined and would be difficult to replicate. Thus, there is a need for protocols to be published in detail, in order to use them in research and clinic. Technology tools are also highly recommended, particularly for home use, as patients may be more adherent and motivated to practice at home.^59^

Outcome selection is crucial to consider while designing studies establishing the efficacy of interventions.^60,61^ It has been recommended that the outcomes are selected to reflect the nearest goal to an intervention.^21^ In exercise interventions, improvements in muscle function (e.g. force generation) and motor skill (e.g. coordination) are typically identified as the most direct goals.^62^ In this review, we found that despite all studies having strength as an intervention goal, only half used strength outcomes. Similarly, range of motion, speed and coordination, as goals, were rarely linked to appropriate outcomes. Most studies focused solely on global function outcomes (i.e. Penetration-Aspiration Scale).^32,36,39,41,47,57^ In these cases, it was difficult to infer the specific physiological changes that led to the improvement in function. Changes that were seen in functional outcomes could be attributed to other factors such as compensatory maneuvers.^63,64^ Thus, the use of muscle and movement measures is highly recommended to improve our understanding of the mechanisms that drive changes in motor ability following interventions.^60^

This review analyzed the reported results based on outcome type and targeted function. Previously published reviews of swallowing exercise interventions found positive evidence of the impact of exercises on muscle function but mixed evidence of the effect of exercises on measures beyond the muscle (i.e. swallowing function).^14,17,65^ However, this review identified several studies with swallowing function measures showing large treatment effects. Nevertheless, this finding still failed to indicate that the exercises were effective because, as mentioned above, the reviewed studies rarely linked muscle/ movement and function outcomes to establish that the improvements in patient functioning were due to intervention.

In agreement with one previous review on facial paresis^13^ and sleep breathing disorder,^12,56^ this review showed that exercises can be highly promising in treating these impairments. All types of measures (including movement measures), albeit in a small number of studies, improved these functions. In contrast, in speech studies, the only measure that had a large treatment effect was maximum isometric muscle pressure. The speech function outcomes (e.g. sentence intelligibility) were not affected. This was not surprising—the use of exercises to improve speech remains controversial as muscle engagement in speech requires speed, high precision, and coordination over strength,^10,11,66^ but the specific exercises and measures targeting and reflecting these types of outcomes are lacking.^11^

Our results suggested that it might be beneficial to consider establishing the effect of exercises across functions as many patients present after stroke with co-occurring impairments.^3,18^ In a clinical setting, speech-language pathologists typically address swallowing and speech disorders; sleep-breathing disorders are managed by respiratory therapists, whereas facial impairments are in the scope of physical or occupational therapists.^5,67,68^ Analyzing the literature across various functions, supported by different rehabilitation disciplines, contributes to advancing a multidisciplinary view of post-stroke care.^69^

This review is not without limitations. First, the elimination of risk of bias and quality assessment in scoping review methodology might have overestimated the actual intervention effects. To avoid deviation from the true effects, the results of each study were reported separately via the forest plot, without conducting quantitative analysis across studies. Second, a single reviewer for abstract/title screening might have increased the possibility of missing relevant studies. To minimize this risk, the reference lists in the included studies and recent reviews were manually searched. Third, the literature examining oral and pharyngeal motor exercises in the context of facial expression, speech, and obstructive sleep apnea remains limited, and the current results might be more relevant only to designing dysphagia interventions.

In conclusion, this scoping review indicated that exercises have been used to restore various oral and pharyngeal muscles and functions. It has also provided insights regarding the appropriate choice of outcome measures. Since post-stroke patients vary in stroke lesions and, consequently, motor impairment (e.g. muscle weakness vs muscle spasticity),^70^ describing the exercise intervention goals with respect to both function and muscle/movement outcomes is essential. Clinicians and researchers may use information in this review when selecting protocols and outcome measures in post-stroke trials. The review also suggested that a single intervention addressing an underlying impairment (e.g. weakness) may affect performance across different functions (e.g. swallowing and sleep-breathing), supporting a multidisciplinary approach toward the development of post-stroke rehabilitations.

**Clinical messages**

- An explicit description of intervention goals, protocols, and outcome measures is needed when developing and publishing the results of oral and pharyngeal exercise interventions.
- A multidisciplinary collaborative view should be adopted in research and practice of evaluating and implementing oral and pharyngeal exercise interventions.

## Supplemental Material

sj-pdf-1-cre-10.1177_02692155221141395 - Supplemental material for The role of oral and pharyngeal motor exercises in post-stroke recovery: A scoping review Click here for additional data file. Supplemental material, sj-pdf-1-cre-10.1177_02692155221141395 for The role of oral and pharyngeal motor exercises in post-stroke recovery: A scoping review by Reeman Marzouqah, Anna Huynh, Joyce L Chen, Mark I Boulos and Yana Yunusova in Clinical Rehabilitation

sj-pdf-2-cre-10.1177_02692155221141395 - Supplemental material for The role of oral and pharyngeal motor exercises in post-stroke recovery: A scoping review Click here for additional data file. Supplemental material, sj-pdf-2-cre-10.1177_02692155221141395 for The role of oral and pharyngeal motor exercises in post-stroke recovery: A scoping review by Reeman Marzouqah, Anna Huynh, Joyce L Chen, Mark I Boulos and Yana Yunusova in Clinical Rehabilitation
