# Supplementary material for: The role of oral and pharyngeal motor exercises in post-stroke recovery: A scoping review
Source: Clin Rehabil. 2022 Nov 25;37(5):620–35. doi: 10.1177/02692155221141395 (PMC10041576; doi:10.1177/02692155221141395)
Supplement: sj-pdf-1-cre-10.1177_02692155221141395 - Supplemental material for The role of oral and pharyngeal motor exercises in post-stroke recovery: A scoping review [file sj-pdf-1-cre-10.1177_02692155221141395.pdf]

Supplementary File. 1 Example of a search strategy implemented for Ovid Medline

1. exp Cerebrovascular Disorders/,

2. stroke*.tw,kf.,

3. exp stroke rehabilitation/,

4. 1 or 2 or 3,

5. exp myofunctional therapy/ or exp speech therapy/ or breathing exercises/ or exp exercise movement technique/,

6. ((Oral or oro*) adj4 (motor or face or facial or pharyn* or strength*)).mp. and (exercise* or train* or intervention or rehab* or stimulation or therap*).tw,kf.

7. exp exercise therapy/,

8. 5 or 6 or 7,

9. exp Deglutition Disorders/ or exp eating/,

10. exp verbal behavior/ or exp speech/ or exp communication disorders/,

11. exp Muscle, Skeletal/,

12. exp sleep apnea syndromes/ or exp mouth breathing/ or exp snoring/,

13. (sleep apnea or snoring or sleep breathing disorder).tw,kf.,

14. (swallow* or deglutition or dysphagia).tw,kf.,

15. (dysarthria or articulation or speech or phonation).tw,kf.,

16. (tongue or lip* or face or mouth or palate or jaw or cheek*).tw,kf.,

17. 9 or 10 or 11 or 12 or 13 or 14 or 15 or 16,

18. 4 and 8 and 17
